# Genome-scale CRISPR-Cas9 knockout screening in hepatocellular carcinoma with lenvatinib resistance

**DOI:** 10.1038/s41420-021-00747-y

**Published:** 2021-11-18

**Authors:** Yonggang Lu, Haoming Shen, Wenjie Huang, Sha He, Jianlin Chen, Di Zhang, Yongqi Shen, Yifan Sun

**Affiliations:** 1 Department of Hepatobiliary Surgery, Affiliated Liutie Central Hospital of Guangxi Medical University, Guangxi, China; 2 Department of Clinical Laboratory, Hunan Cancer Hospital & The Affiliated Cancer Hospital of Xiangya School of Medicine, Central South University, Hunan, China; 3 Department of Clinical Laboratory, Affiliated Liutie Central Hospital of Guangxi Medical University, Guangxi, China; 4 Department of Clinical Laboratory, The Third Xiangya Hospital of Central South University, Hunan, China; 5 Department of Oncology, Affiliated Liutie Central Hospital of Guangxi Medical University, Guangxi, China

**Keywords:** Cancer, Biomarkers, Medical research

## Abstract

Lenvatinib is the first target drug approved for advanced hepatocellular carcinoma (HCC). However, the development of drug resistance is common, and the mechanisms of lenvatinib resistance and resistant targets in HCC are poorly understood. By using CRISPR/Cas9 library screening, we screened out two key resistance genes, neurofibromin 1(NF1), and dual specificity phosphatase 9 (DUSP9), as critical drivers for lenvatinib resistance in HCC. With RNAi knockdown and CRISPR/Cas9 knockout models, we further clarified the mechanisms by which NF1 loss reactivates the PI3K/AKT and MAPK/ERK signaling pathways, while DUSP9 loss activates the MAPK/ERK signaling pathways, thereby inactivating FOXO3, followed by degradation of FOXO3, finally induced lenvatinib resistance. We also screened out trametinib, a small molecule pathway inhibitor for MEK, that can be used to reverse resistance induced by NF1 and DUSP9 loss in HCC cells. Trametinib was still able to halt HCC growth even when NF1 was knocked out in mice. Collectively, the findings indicate that NF1 and DUSP9 takes critical role in lenvatinib resistance and may be novel specific targets and predictive markers for lenvatinib resistance in HCC.

## Facts

It is imperative to explore the precise molecular mechanisms of chemoresistance and to develop effective targeted therapies to diminish HCC chemoresistance. Lenvatinib has been found to be resistant in HCC, however, the mechanisms of lenvatinib resistance and resistant targets in HCC, are poorly understood. Thus, understanding the molecular mechanisms that lead to lenvatinib chemoresistance is essential to developing more effective treatments against HCC.

## Open questions

1. Can lenvatinib resistance genes in HCC be screened using CRISPR/cas9 library?
2. What signaling pathways do the screened drug resistance genes lead to drug resistance?
3. Is there a suitable pathway inhibitor to reverse drug resistance?

## Introduction

Primary liver cancer is one of the most common malignant tumors and the fourth leading cause of cancer-related mortality worldwide [1]. Although liver cancer can be classified into several different types, hepatocellular carcinoma (HCC) is the most common primary malignant liver tumor in adults [2]. The best choice of treatment for HCC, but not for advanced HCC patients, is surgical resection. However, typical symptoms are absent in the early stage of HCC, and specific diagnostic biomarkers for HCC are deficient. As a result, HCC is usually diagnosed at a relatively late stage, with patients thereby losing the opportunity to receive radical surgery. In advanced or intermediate stages, when chemoembolization is no longer indicated, systemic treatment represents the standard therapy for HCC. Molecular targeted drugs are among the main treatment options for advanced-stage HCC. At present, four orally administered small-molecule multikinase kinase inhibitors (MKIs)—namely, sorafenib, lenvatinib, regorafenib, and cabozantinib—have been approved in Europe for advanced HCC indication. Sorafenib is the first target drug approved for advanced HCC, but only ~30% of liver cancer patients are sensitive to sorafenib, and most patients will develop drug resistance after an average of 6 months [3]. It can be seen that the sensitivity of patients to drugs limits the clinical application of targeted drugs, and drug resistance leads to treatment failure.

In 2018, lenvatinib (III) was approved by the FDA as a first-line treatment for advanced and unresponsive patients with HCC. Lenvatinib is a small molecule multi-target RTK (receptor tyrosine kinase) inhibitor. It mainly acts on VEGFR (1-3), RET, FGFR (1-4), c-kit, PDGFR-α, and PDGFR-β. It can inhibit RTKs related to pathological angiogenesis, tumor growth, and cancer progression and has been used before for the treatment of progressive radioiodine refractory differentiated thyroid cancer before [4, 5]. Excitingly, lenvatinib was shown to be noninferior to the standard-of-care treatment, sorafenib, in terms of the primary outcome of overall survival (OS) in unresectable HCC patients in a randomized phase III non-inferiority (REFLECT) trial [6]. Although lenvatinib is clinically efficacious, acquired resistance to it is inevitable because under the selective pressure of molecular targeted therapy, drug resistance mutations commonly occur. Indeed, lenvatinib has been found to be resistant in some tumors [7, 8] as well as HCC [9]. Recent studies have revealed the mechanisms of sorafenib resistance in HCC and found many biomarkers that can predict sorafenib resistance [10]. However, the mechanisms of lenvatinib resistance and resistant targets in cancers, especially in HCC, are poorly understood. This will affect clinical decisions and limit the correct use of lenvatinib in HCC.

It is imperative to explore the precise molecular mechanisms of chemoresistance and to develop effective targeted therapies to diminish HCC chemoresistance. For example, targeting PHGDH is an effective approach for overcoming sorafenib drug resistance in HCC [11]. SHP2 inhibitor shp099 can eliminate sorafenib resistance in hepatoma cell lines by blocking the Ras/MEK/ERK negative feedback mechanism [12]. Yet little is known about the synergistic effect of small molecule pathway inhibitors and lenvatinib in HCC. Thus, understanding the molecular mechanisms that lead to lenvatinib chemoresistance is essential to developing more effective treatments against HCC.

There are many methods for screening resistance genes, including cDNA libraries, RNAi screening and the CRISPR/Cas9 library. RNAi screening using the shRNA library to down-regulate specific target genes is a well-established method for loss-of-function screening. Compared with RNAi screening, the CRISPR/Cas9 knockout library provides a higher screening sensitivity since incomplete knockdown by RNAi sometimes may not be sufficient to generate the loss-of-function phenotype [11]. In this study, we attempted to combine the CRISPR library screening strategy with RNA sequencing technology to explore the critical genes and potential mechanism for lenvatinib resistance in HCC. We screened out two key resistance genes, neurofibromin 1(NF1) and dual specificity phosphatase 9 (DUSP9) and confirmed their mechanism of inducing lenvatinib resistance by activating the PI3K/AKT and MEK/ERK signaling pathways in vivo and in vitro. We also screened out trametinib, a small molecule pathway inhibitor that can be used to reverse lenvatinib resistance in HCC. This is of great significance for formulating a lenvatinib combined therapy strategy, improving drug resistance, and prolonging the survival of HCC patients.

## Results

### CRISPR library screening results of lenvatinib in Huh7 cells

**Fig. 1:**
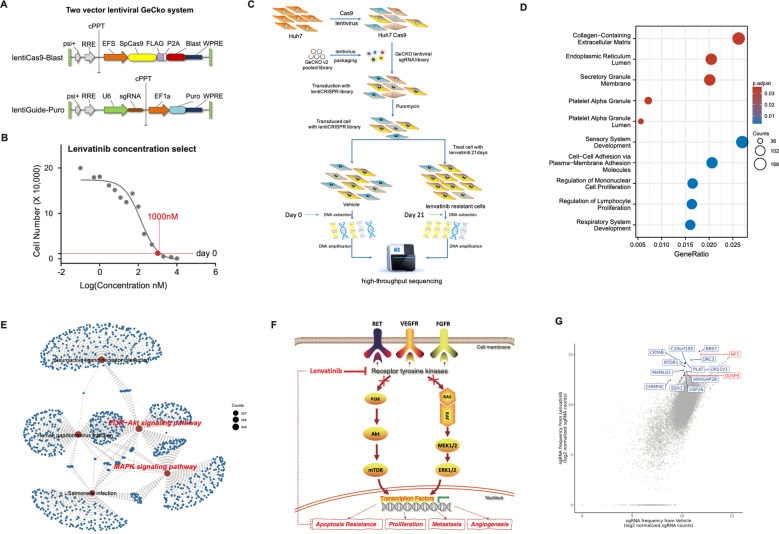
Schematic and results of functional screening by sgRNA library. **A** Structure of Two vector lentiviralGeCKO system. **B** The best lenvatinib concentration for treatment. Using different lenvatinib concentrations to treat Huh7 cells, after 21 days, the number of surviving cells in 1000 nM group was the closest to that after zero days. **C** Schematic diagram illustrates the workflow of genome-wide CRISPR/Cas9 knockout library screening. Human genome-wide CRISPR/Cas9 knockout library (GeCKO v2A) was packed into lentiviral particle and transduced into Cas9-overexpressing Huh7 cells at low multiplicity of infection (MOI = 0.5). The sgRNA transduced cells were selected by puromycin for 7 days. The cells were then divided into vehicle and lenvatinib groups for culture. After 21 days, lenvatinib-resistant cells were enriched (Day 21), and then cells of vehicle group (Day 0) and lenvatinib group (Day 21) were collected for genomic DNA extraction and high-throughput sequencing. **D** GO analysis showed that the candidate 1261 genes are involved in cell proliferation. The red dots indicated the top 5 enriched BP (biological process) of GO analysis. The blue dots indicated the top 5 enriched CC (cellular component). **E** KEGG analysis showed that the candidate 1261 genes are involved MAPK/ERK and PI3K/AKT signaling pathways. Only showed the top5 signaling pathways. **F** Diagram of the mechanism of lenvatinib in cancer. Lenvatinib exerts its anticancer effect by inhibiting PI3K/AKT and MEK/ERK signaling pathways through target receptor tyrosine kinase. **G** The potential 14 genes induced lenvatinib resistance according to bioinformatics analysis and gene function.

To identify critical genes that modulate the response to lenvatinib (HY-10981, MCE) resistance in HCC, we performed a genome-wide CRISPR/Cas9 knockout library screening using HCC cell line Huh7. We hypothesized that knockout of lenvatinib-sensitive genes would make HCC cells resistant to lenvatinib-induced cell death or proliferation suppression. That is, cells carrying sgRNA targeting lenvatinib-sensitive genes will be positively selected in the mutant cell pool, and their corresponding sgRNA will also be enriched in the library, which can be determined by high-throughput sequencing. We found that when the lenvatinib concentration was 1000 nM, the number of surviving cells after 21 days of treatment was the closest to that after 0 days. Therefore, we used 1000 nM lenvatinib to treat the cells after 7 days of puromycin screening to obtain lenvatinib-resistant cells (Fig. 1B). Figure 1C displayed a schematic of lenvatinib-resistant Huh7 cells construction for high-throughput sequencing analysis. Genomic DNA was isolated on Day 21, and sgRNAs were quantified by sequencing. SgRNAs that were enriched in the Day 21 samples compared to the Day 0 samples were then identified if there was a cutoff of log2 fold change of at least 2. From this CRISPR/Cas9 knockout library screening, we identified a subset of sgRNAs targeting 1261 genes that were significantly enriched (Fold change >2), indicating that knockout of these genes might be potential drivers for lenvatinib resistance (Fig. S1). GO analysis revealed that these genes were predominantly enriched in cell killing (GO:0031341), proliferation (GO:0032944), and adhesion (GO:0098742) (Fig. 1D and Table S4). KEGG pathway analysis predicted that these genes featured enrichment for the MAPK/ERK and PI3K/AKT signaling pathways (Fig. 1E). Indeed, lenvatinib exerts its anticancer effect by inhibiting PI3K/AKT and MEK/ERK signaling pathways (Fig. 1F). Among the top of the gene list, we selected a set of 14 sgRNAs for further analysis based on their functional annotations, such as NF1, a negative regulator of Ras/MAPK and PI3K/AKT signaling; DUSP9, a dual-specificity phosphatase that inhibits ERK; and PLAT (plasminogen activator), a gene enzyme that plays a role in cell migration (Fig. 1G).

### NF1 and DUSP9 loss induce lenvatinib resistance

**Fig. 2:**
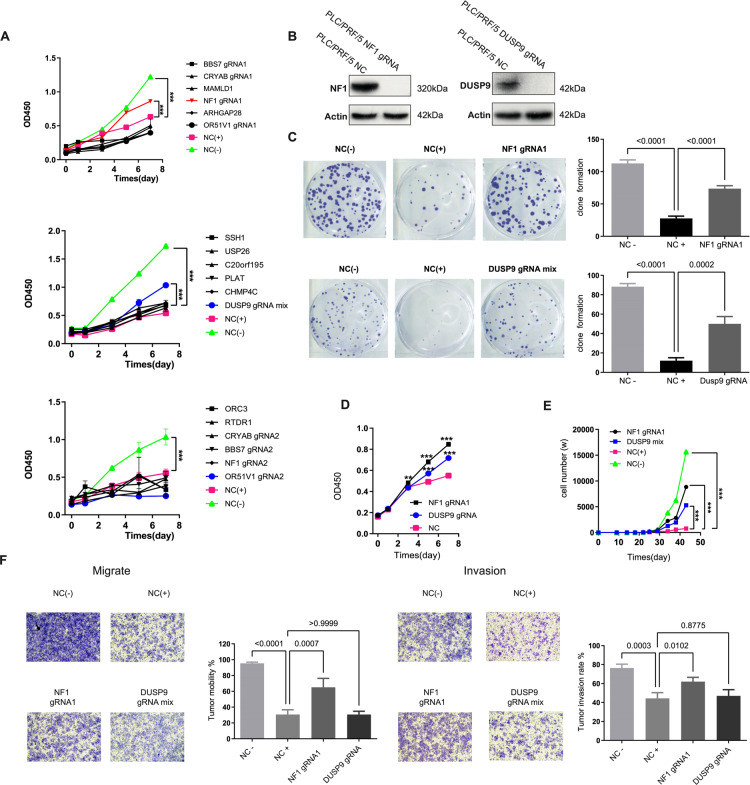
Depletion of NF1 or DUSP9 can reverse the inhibitory effect of lenvatinib on Huh7 cell. **A** Cell viability assessed by CCK8 assay for 14 candidate genes. Depletion of NF1 and DUSP9 showed a significant proliferative advantage relative to the other candidate genes under lenvatinib treatment. The 14 candidate genes were separated as three groups (There are two sgRNA for BBs7, NF1, CRYAB, OR51V1, respectively. NC-, Negative control, without drug.NC + , Negative control, under drug treatment). **B** NF1 and DUSP9 knockout expression levels were confirmed by western blotting in Huh7 cell. Expression of NF1 or DUSP9 is undetectable in Huh7 cell after knocked out by NF1 or DUSP9 sgRNA, respectively. **C** Clone formation capacity of Huh7 cells was assessed by the clone formation assay. Lenvatinib significantly inhibited Huh cells proliferation compared with NC-, however, the number of clones significantly increased again after NF1 or DUSP9 knockout. **D** CCK8 assay for NF1 and DUSP9 knockout. **E** Cell number counting for NF1 and DUSP9 knockout. **F** Transwell migration assay and transwell invasion assay were performed in Huh 7 cell. Error bars represent mean ± SEM for triplicate experiments, *P < 0.05, **P < 0.01, ***P < 0.001 or the values were shown in the figures.

To verify the functions of the 14 candidate genes, we first performed cell viability and colony formation assays using Hun7 cells stably expressing Cas9 that were infected with lentivirus expressing specific gRNA and selected by antibiotics. Clonogenic assay validated MAMLD1, NF1 sgRNA1, SSH1, USP26, PLAT, CHMP4C, DUSP9, and OR51V1 with a more obvious clonogenic advantage Fig. S2; this revealed these eight genes that, when knocked out, mediated lenvatinib resistance. For investigation of cell proliferation, experiments were performed in three groups. As shown in Fig. 2A, lenvatinib was added to sgRNA stable cell lines expressing six genes (USP26, SSH1, C20orf195, PLAT, CHMP4C, DUSP9). The DUSP9 group showed a significant proliferative advantage relative to the NC+ and various others, indicating that DUSP9 knockout may have contributed to lenvatinib resistance. In the same way, NF1 showed the same trend in another group (BBS7, CRYAB, MAMLD1, NF1 gRNA1, ARHGAP28, OR51V1). However, ORC3, RTDR1, CRYAB gRNA2, BBS7 gRNA2, and OR51V1 gRNA2 showed no significant proliferative advantage over the NC+ , for which NF1 gRNA2 also had no effect. Based on the above results, we have preliminarily concluded that loss of NF1 and DUSP9 in Huh7 cells mediated lenvatinib resistance. Western blotting confirmed that it is not expressed after NF1 and DUSP9 knockout in Huh7 cells (Fig. 2B). In repeated cell viability and colony formation experiments (Fig. 2C, D), lenvatinib has demonstrated the ability to significantly inhibit cell proliferation, and knockout of NF1 and DUSP9 significantly blocks the ability of lenvatinib to inhibit cell proliferation as well as the effect on cell number (Fig. 2E). Its invasion and migration capacities have also been significantly improved after knockout of NF1 in Huh7 cells compared with cells subject to lenvatinib treatment alone. However, knockout of DUSP9 did not affect cell migration and invasion (Fig. 2F). Additionally, we observed that knockout of NF1 or DUSP9 had no apparent effects on cell apoptosis (Fig. S3A) and cycle (Fig. S3B).

**Fig. 3:**
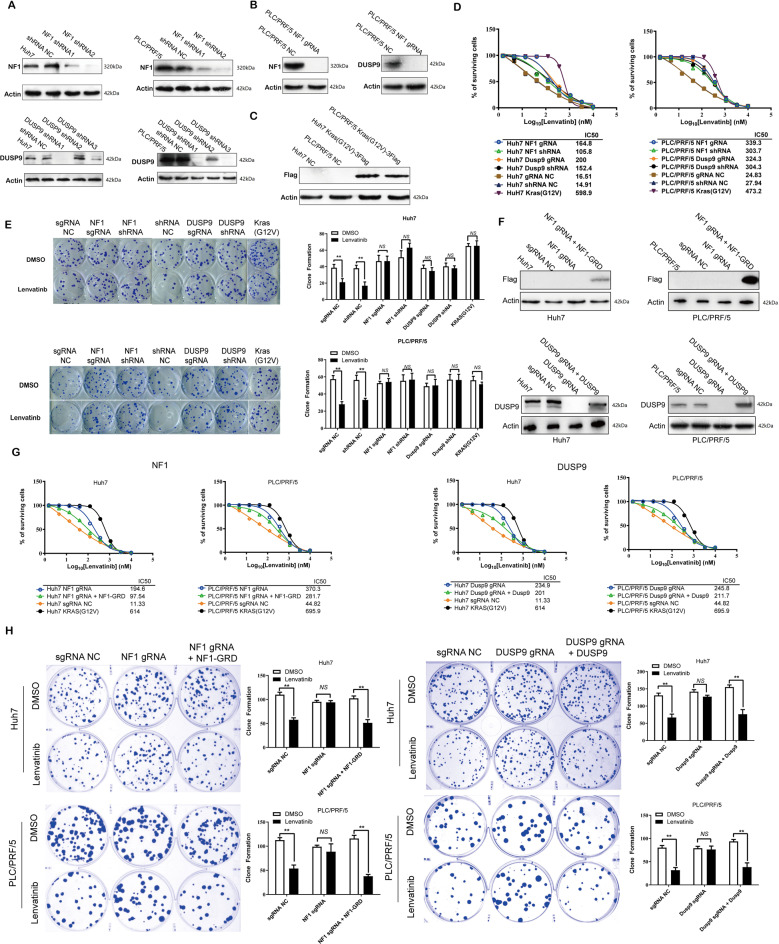
NF1 and DUSP9 loss induce lenvatinib resistance. **A** Changes in NF1 and DUSP9 expression upon shRNA knockdown were analyzed by Western blotting. NF1 shRNA2 and DUSP9 shRNA1 have the best interference effect, and can be used as NF1 and DUSP9 knockdown interference cell line for further experiments. **B** NF1 and DUSP9 knockout expression levels were confirmed by western blotting in PLC/PRF/5 cell. Expression of NF1 or DUSP9 is undetectable in PLC/PRF/5 cell after knocked out by NF1 or DUSP9 sgRNA, respectively. **C** KRAS(G12V) overexpression levels were analyzed by anti-Flag blots (WB) in Huh7 and PLC/PRF/5 cells. DNA from SW480 cell line (KRAS G12V mutation) served as a positive control. **D** Comparison of IC50 based on MTT assay in Huh7 and PLC/PRF/5 cells. IC50 was significantly increased after NF1 and DUSP9 were knocked out or knocked down compared with those in NC. **E** Clone formation assay in t Huh7 and PLC/PRF/5 cells using gene knockout or knockdown assays. All NF1 and DUSP9 knockout or knockdown clones showed markedly enhanced colony formation capacities from those in control cells. **F** NF1 and DUSP9 overexpression levels were confirmed by western blotting in Huh7 and PLC/PRF/5 cells. The repression of NF1 and DUSP9 by sgRNA knocked out could be reversed after NF1 and DUSP9 overexpression. **G** MTT assay showed that IC50 value after NF1 and DUSP9 overexpression was significantly decreased again compared with those in NF1 and DUSP9 knockout or knockdown cells. **H** NF1 and DUSP9 overexpression reversed the colony formation capacities enhanced by NF1 and DUSP9 loss. Error bars represent mean ± SEM for triplicate experiments, *P < 0.05, **P < 0.01, ***P < 0.001, ns no significantly changed.

To further consolidate our CRISPR/Cas9 knockout library screening result, we constructed knockout and RNAi cell lines of NF1 and DUSP9 in two HCC cells, Huh7 and PLC/PRF/5 (Fig. 3A, B), while the Kras (G12V) overexpression cell line served as a positive control (Fig. 3C). We then performed MTT assays and clonogenic cell survival assays to further confirm whether loss of NF1 and DUSP9 causes lenvatinib resistance in cancer cells. After using sgRNA to knockout NF1, the IC50 value of the Huh7 cells increased 12-fold (from 16.51 to 164.8), and 13-fold (from 24.83 to 339.3) in PLC/PRF/5 cells. After DUSP9 was knocked out by sgRNA in Huh7 cells, IC50 increased from 16.51 to 200.0 and from 24.83 to 303.7 in PLC/PRF/5 cells. After silencing the target gene with shRNA in two cell lines, the IC50 of lenvatinib resistance increased by 7–12-fold (Fig. 3D). After NF1 and DUSP9 knockout or knockdown, the colony-forming ability of Huh7 and PLC/PRF/5 cells under lenvatinib pressure was not significantly changed compared with the subgroup not medicated with lenvatinib (Fig. 3E). Furthermore, we constructed overexpression of NF1 and DUSP9 for rescue experiments, as shown in Fig. 3F, sgRNA deletion of NF1 and DUSP9 was reversed by NF1-GRD and DUSP9 overexpression. The results of MTT assay showed that overexpression of NF1 or DUSP9 led to a decrease in IC50 values, and the most pronounced effect was seen after overexpression of NF1 (Fig. 3G). In both cell lines, clonogenic capacity increased significantly under lenvatinib pressure compared with lenvatinib-nonmedicated groups after NF1 or DUSP9 knockout, and clonogenic capacity decreased again after overexpression of NF1 or DUSP9 (Fig. 3H). These results indicated that NF1-GRD and DUSP9 overexpression reversed the inhibition effect towards treatment with lenvatinib of sgRNA-mediated knockout in HCC cells.

Taken together, we identified several novel factors that have the capacity to influence lenvatinib resistance through the screening process described above. Moreover, NF1 and DUSP9 have been verified as two critical genes that participate in lenvatinib resistance in HCC and are implicated in essential processes, including cell proliferation, invasion, and migration.

### NF1 and DUSP9 loss activate PI3K/AKT and MAPK/ERK signaling pathways

**Fig. 4:**
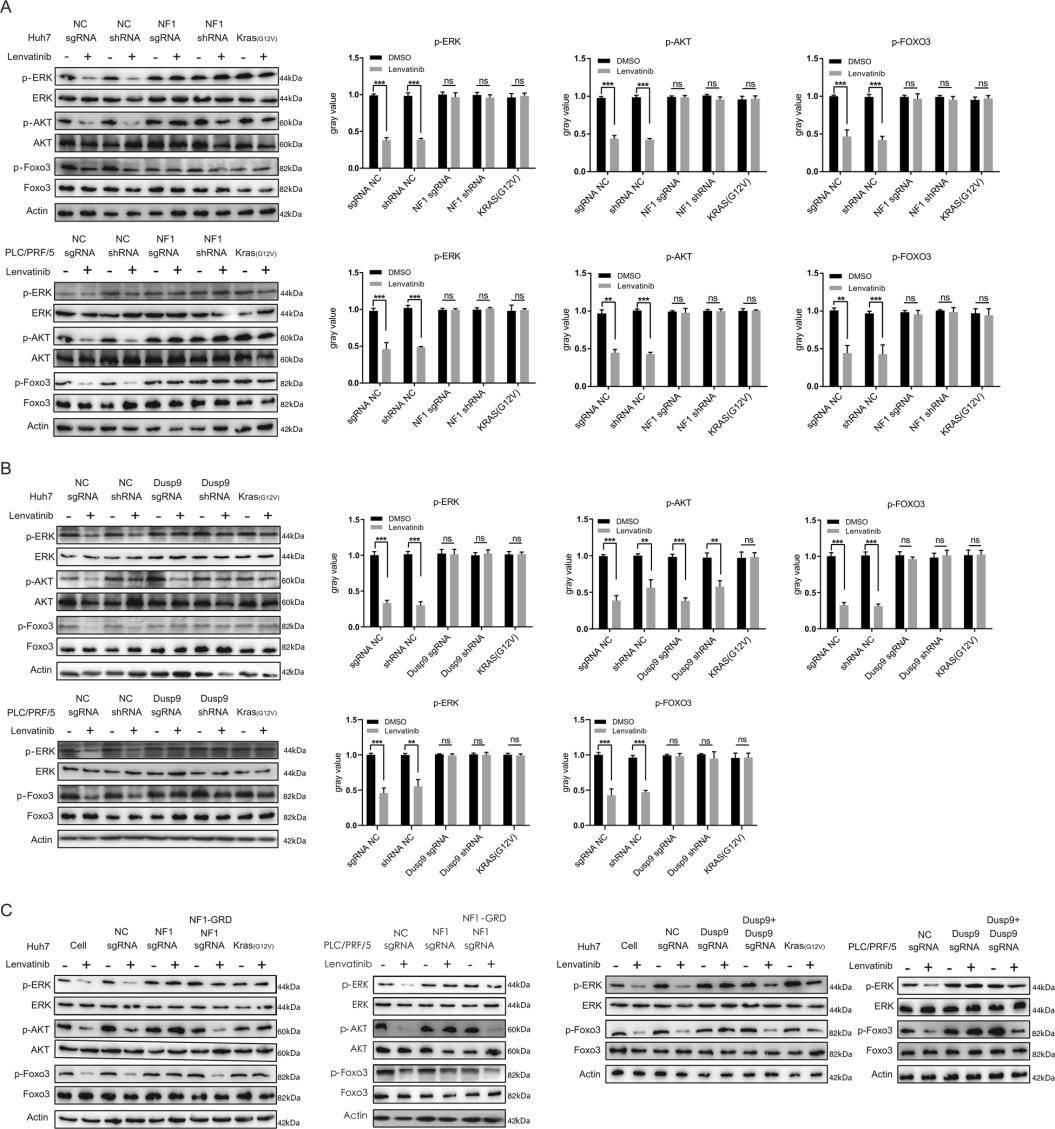
NF1 and DUSP9 loss reactivate PI3K/AKT and MAPK/ERK signaling pathways. The expression levels of phosphorylated AKT, ERK, FOXO3 were assayed by WB analysis in Huh7 and PLC/PRF/5 cells after NF1 (**A**) and DUSP9 (**B**) were knocked out or knocked down. The expression levels of phosphorylated AKT, ERK, FOXO3 were significantly increased after NF1 were knocked out or knocked down, however, depletion of DUSP9 only increased the expression levels of phosphorylated ERK and FOXO3. **C** NF1 and DUSP9 overexpression reversed the expression levels of phosphorylated AKT, ERK, FOXO3 enhanced by NF1 and DUSP9 loss. Error bars represent mean ± SEM for triplicate experiments, *P < 0.05, **P < 0.01, ***P < 0.001, ns no significantly changed.

As a small molecule multi-target RTK inhibitor, lenvatinib suppresses the PI3K/AKT/mTOR and Ras/Raf/MAPK/ERK signaling pathways, which are key signaling pathways that regulate cell proliferation and differentiation (Fig. 1F). Therefore, we performed a simple verification experiment using western blotting. Indeed, knockout of NF1 by sgRNA increased AKT and ERK phosphorylation, which was inhibited by lenvatinib. However, sgRNA-mediated DUSP9 deletion increased ERK but not AKT phosphorylation in Huh7 cells (Fig. S4). In order to further test our hypothesis, AKT and ERK phosphorylation was examined by western blotting for all models. FOXO3 phosphorylation was also examined as previous studies have established that activation of the PI3K/Akt and MAPK/ERK pathways can lead to FOXO3 phosphorylation [13, 14]. When lenvatinib was added to Huh7 or PLC/PRF/5 control cells, ERK, AKT, and FOXO3 phosphorylation were inhibited, lenvatinib could not inhibit the phosphorylation of ERK, AKT, and FOXO3 after NF1 was knocked down by shRNA or knocked out by sgRNA (Fig. 4A). Consistently, when DUSP9 was knocked down by shRNA or knocked out by sgRNA, the phosphorylation levels of ERK and FOXO3 were also increased, but no change was observed for the phosphorylation levels of AKT in the presence of lenvatinib (Fig. 4B). Moreover, all these changes could also be reversed by NF1 and DUSP9 overexpression (Fig. 4C). Taken together, these data suggested that loss of NF1 could activate the PI3K/AKT and MAPK/ERK signaling pathways to promote lenvatinib resistance in HCC, while loss of NF1 DUSP9 could activate the MAPK/ERK but not the PI3K/AKT pathway to promote lenvatinib resistance, and the potential downstream regulatory target gene is FOXO3.

### Trametinib is still sensitive to lenvatinib resistance due to NF1 and DUSP9 gene deletion

**Fig. 5:**
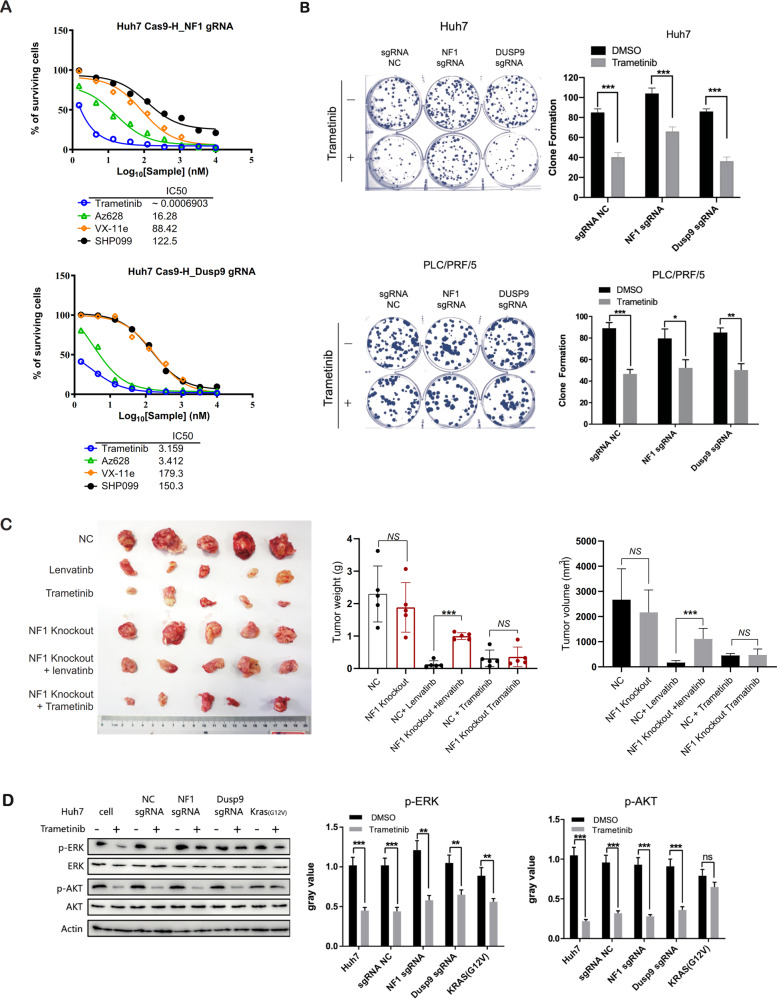
Trametinib sensitize HCC treatment with NF1 loss through ERK and AKT reactivation. **A** Trametinib was the most sensitive drug for the loss of Huh7 NF1 or DUSP9 cells. The IC50 value of trametinib toward the Huh7 NF1 sgRNA or DUSP9 sgRNA cells was significantly lower than that of Az628, VTX-11e, and SHP009. **B** Trametinib could inhibit colony formation capacities from those in NF1 or DUSP9 knockout cells. **C** Trametinib was still able to halt HCC growth when NF1 was knocked out. The tumor weight and volume were significantly decreased after lenvatinib or trametinib treatment. However, the tumor weights or volumes of NF1 sgRNA cell-injected mice were significantly higher than those of tumors without NF1 knockout cells after lenvatinib treatment, and trametinib treatment did not show any difference in both. **D** The ERK and AKT phosphorylation was sustained in the presence of trametinib in Huh7 cells. Error bars represent mean ± SEM for triplicate experiments, *P < 0.05, **P < 0.01, ***P < 0.001, ns no significantly changed.

**Fig. 6:**
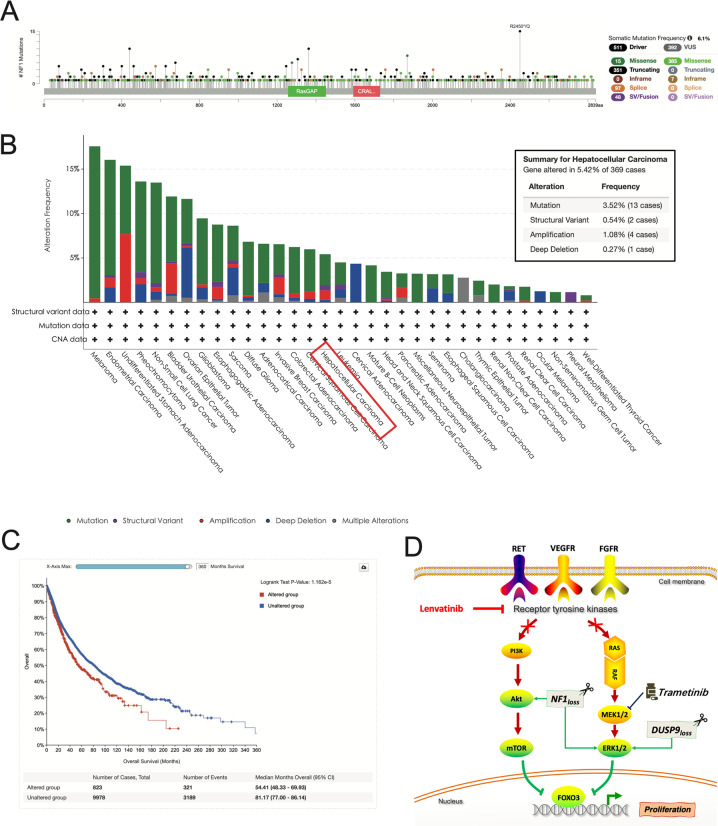
NF1 alterations is common in cancers. **A** NF1 alterations in cancers. NF1 somatic mutation frequency showed in cBioPortal cancer datasets was 6.1%. **B** NF1 altered in multiple cancers. The NF1 altered in 5.42% of 392 HCC cases. **C** NF1 altered patients had poorer outcomes compared to unaltered patients. **D** A schematic depicting the suggested mechanism. Lenvatinib exerts its anticancer effect by inhibiting PI3K/AKT and MEK/ERK signaling pathways through target receptor tyrosine kinase. NF1 loss can active Akt and ERK phosphorylation, and DUSP9 loss can active ERK phosphorylation, further induces FOXO3 phosphorylation, leading to degradation of FOXO3, finally resulting in the progression of HCC. Trametinib, a small-molecule protein kinase inhibitors target MEK, can still inhibit the progression of HCC even NF1 loss by inhibited Akt and ERK phosphorylation.

Having established that the loss of NF1 could active PI3K/AKT and MAPK/ERK, and that the loss of DUSP9 could activate the MAPK/ERK signaling pathway, we sought to determine whether NF1 or DUSP9 knocked out could also affect sensitivity to related signaling pathway inhibitors. Small-molecule protein kinase inhibitors such as the MEK inhibitor trametinib (Selleck), RAF inhibitor Az628 (Selleck), ERK inhibitor VTX-11e (Selleck), and RAS inhibitor SHP009 (Selleck) were selected as drugs to treat Huh7 cells, and IC50 values were determined using MTT assay. The IC50 value of trametinib toward the Huh7 NF1 sgRNA or DUSP9 sgRNA cells was significantly lower than that of Az628, VTX-11e and SHP009 (Fig. 5A), suggesting that trametinib is the most sensitive drug for the loss of Huh7 NF1 or DUSP9 cells. We next tested the functional ability of trametinib treatment in Huh7 and PLC/PRF/5 NF1 or DUSP9 sgRNA cells using clonogenic assays. Compared with the group receiving no drug treatment, Trametinib treatment significantly inhibited cell proliferation, even when NF1 or DUSP9 were knocked out by sgRNA (Fig. 5B). Next, we performed subcutaneous injection of Huh7 or Huh7 NF1 sgRNA cells in nude mice. When tumors were palpable, the mice were randomly divided into six groups and were treated with vehicle controls (NC and sgRNA NC, respectively), lenvatinib alone and trametinib alone (Fig. 5C). We found that lenvatinib and trametinib are both capable of efficiently inhibiting tumorigenesis caused by Huh7 cells in mice. However, lenvatinib had a significantly weaker inhibitory effect on tumorigenesis caused by NF1 sgRNA cells, indicating the development of resistance. Excitingly, trametinib was still able to halt HCC growth even when NF1 was knocked out (Fig. 5C). Furthermore, ERK and AKT phosphorylation was also sustained in the presence of trametinib in Huh7 cells (Fig. 6C). These data together confirmed that the MEK inhibitor trametinib could sensitize HCC treatment with NF1 loss through reactivation of ERK and AKT.

### The magnitude of NF1 alterations in human cancers

To understand the magnitude of NF1 alterations in human cancers, we used the cBio Cancer Genomics Portal (cBioPortal: http://www.cbioportal.org) to interrogate cancer genomic data for alterations of NF1 in large numbers of tumor samples from cancer studies.NF1 somatic mutation frequency showed in cBioPortal cancer datasets was 6.1%, and there are 903 mutations in patients with multiple samples (Fig. 6A). Somatic mutations in the NF1 gene have been found in human tumors, amongst which nonsense mutations, splice site mutations, missense changes, and frameshift indels were present, and the NF1 altered in 5.42% of 392 HCC cases (Fig. 6B). In TCGA PanCancer Atlas Studies with 10,953 patients/10,967 samples, there are 839 samples have alterations in the NF1 gene. The mean survival time in the gene altered group was 54.41 months, whereas the mean survival time in the unaltered group was 81.17 months (Fig. 6C). These data indicated that NF1 loss is common in cancers and promote the progression of cancers, which confirmed the effect of NF1 loss in lenvatinib resistance in HCC.

## Discussion

Drug resistance is a major hurdle in the treatment of cancer. An important goal in HCC therapy is to overcome drug resistance, either by deciphering resistance mechanisms or by identifying new drugs with synergistic effects when administered with current treatments. Therefore, identifying the underlying mechanism and discovering new therapeutic strategies for chemoresistance is very important. In this study, we performed a genome-wide screening in HCC cells treated with and without lenvatinib and identified NF1 and DUSP9 loss-induced lenvatinib resistance in HCC. As shown in Fig. 6D, we found the mechanism behind this: the loss of NF1 and DUSP9 can activate the PI3K/AKT and MAPK/ERK signaling pathways, thereby inactivating FOXO3, followed by degradation of FOXO3; this results in the progression of HCC, which is inhibited by lenvatinib. We further found that Trametinib would still be expected to be sensitive to the loss of NF1. These findings are important in several senses: first, it is a discovery that NF1 and DUSP9 are two key resistance genes for lenvatinib treatment, and that these two genes are potential therapeutic targets and valuable predictors of lenvatinib resistance in HCC; second, it is a demonstration that PI3K/AKT and MAPK/ERK are the resistance signaling pathways of NF1 and DUSP9; third, it provides evidence that trametinib might synergize with lenvatinib to treat HCC more effectively.

Lenvatinib is a multi-kinase inhibitor that can inhibit the rearrangement of the transfected oncogene and proto-oncogene, such as the c-kit gene, and then inhibit tumor proliferation [15]. Lenvatinib inhibits its downstream pathways, including PI3K/AKT and MEK/ERK signaling pathways, by inhibiting kinases. Therefore, we selected target genes that were related to the PI3K/AKT and MEK/ERK signaling pathways. Finally, we found that NF1 deletion induced lenvatinib resistance through phosphorylation of AKT and ERK, and the deletion of DUSP9 did so through phosphorylation of ERK and FOXO3. Previous studies on tumor resistance due to NF1 loss mainly focused on the activation of Ras/RAF kinases, such as the Raf-MEK-ERK pathway [16]. Our results also showed that NF1 deletion reactivated the MAPK/ERK signaling pathway in lenvatinib-inhibited HCC cells, which is consistent with previous results. We also found that NF1 loss activates the PI3K/AKT pathway, whereas DUSP9 loss only activates the MAPK/ERK signaling pathway. DUSP9 loss also resulted in less pronounced effects than NF1 loss. In previous studies, DUSP9 expression was downregulated in tumors and mediated the progression of CRC [17] and breast cancer [18] through the MAPK/ERK signaling pathway, which participated in HCC cell proliferation by regulating glucose metabolism and secretion of inflammatory factors [19]. Taken together, the above studies suggest that therapeutic interventions that increase the expression or activity of DUSP9 may activate antiproliferative signals in malignant cells.

FOXO3, as a tumor inhibitor, is a transcription factor with multiple biological functions, including antioxidant response, longevity, and cell cycle control. FOXO3 Ser294 has been demonstrated to be a target of Ras-MAPK1/3 signaling, which is followed by downregulation of FOXO3 activity; the ERK-phosphorylated FOXO3a degrades via an MDM2-mediated ubiquitin-proteasome pathway [13]. AKT acts downstream of PI3K to regulate many biological processes, such as proliferation, apoptosis and growth [20]. Activated AKT phosphorylates many downstream targets, including FOXO3, followed by nuclear exclusion and degradation of FOXO3, resulting in the progression of diseases [21, 22]. Moreover, FOXO3 is an important target of m6^A^ modification in the resistance of HCC to sorafenib therapy [23]. We speculated, therefore, that activated AKT or ERK phosphorylates many downstream FOXO3, leading to lenvatinib resistance. As expected, in our study, phosphorylated FOXO3 was activated after knockout or knockdown of NF1 and DUSP9, suggesting that NF1 or DUSP9 loss induced FOXO3 degradation via phosphorylation of AKT or ERK. Nevertheless, the detailed mechanism of FOXO3-induced lenvatinib resistance in HCC has yet to be fully elucidated.

Previous studies have shown that targeting drug resistance resulting from gene deletion through its acting signaling pathways, with the corresponding inhibitors, can improve the resistance of targeted drugs [24]. For example, treatment of NF1-deficient lung cancers with map-ERK kinase (MEK) inhibitors restores sensitivity to erlotinib [25]; and combining type II Rafi with an allosteric MEKI reliably prevents and overcomes acquired drug resistance in cancers with NF1 mutations. We found trametinib caused a better killing effect of both NF1 knockout Huh7 cells in animal experiments, illustrating that trametinib can still be used in Lenvatinib-resistant Huh7 NF1 knockout cell lines. Trametinib is a highly selective MAPK kinase (MEK) 1/2 allosteric inhibitor and exerts anticancer activity against a variety of cancers. Trametinib can be used in combination with many drugs to harness a synergistic effect. Early studies proposed that the combination of dabrafenib and trametinib improved anti-tumor activity and survival in BRAF mutant melanoma patients [26], and follow-up research reported that trametinib induced the reduction of DUSP6, while the result of increased p53 phosphorylation synergizes with MDM2 inhibition in cutaneous melanoma [27]. Trametinib also can enhance Mcl1 degradation-mediated apoptosis in combination with TRAIL in colorectal cancer cells [28]. Our study confirmed that trametinib could sensitize HCC to lenvatinib treatment.

## Conclusions

Our study used CRISPR-library screening to systematically identify NF1 and DUSP9 as two critical drivers for lenvatinib resistance in HCC, and further clarified the mechanisms by which NF1 loss reactivates the PI3K/AKT and MAPK/ERK signaling pathways, while DUSP9 loss activates the MAPK/ERK signaling pathways, leading to lenvatinib resistance in HCC. We also screened out trametinib for reversing lenvatinib resistance, which is important for developing lenvatinib combination therapy strategies. In conclusion, our study provides predictors of lenvatinib resistance in HCC, and find trametinib has potential synergistic effect for lenvatinib treatment.

## Methods

### Cell lines and animals

HCC cell lines Huh7, PLC/PRF/5 and Human embryonic kidney 293T (HEK293T) were obtained from the cell bank of the Chinese Academy of Sciences and were authenticated by STR profiling. All cells were cultured under 37 °C with 5% CO2. BALB/c nude mice (5–6 weeks old) were purchased from Shanghai Jihui Laboratory Animal Care, China. The mice were single caged seven days before the start of the experiments. All experimental procedures conformed to the NIH Guide for the Care and Use of Laboratory Animals and were approved by the Ethics Committee of Liuzhou Municipal Liutie Central Hospital.

### Genome-wide CRISPR/Cas9 knockout library screen and sequence

The genome-wide CRISPR-Cas9 gene knockdown screen was accomplished using HuH7 human hepatoma cells and the GeCKOv2 gene knockout library (Addgene, #1000000048). The human GeCKOv2 library consists of two parts, sgRNA libraries A and B, which contain 123,411 sgRNA targeting 19,050 genes and 1000 non-targeted sgRNA as a control. To obtain a cell-efficient gecko library on the lentiviral vector, Lentiviral LentiCas9‐Blast (Addgene plasmid #52962) and LentiGuide‐Puro (Addgene plasmid #52963) single guide RNA plasmids were processed according to Lentiviral CRISPR ToolBox protocol GeCKO (Fig. 1A). A genome-wide CRISPR-Cas9 screen was then performed, as described previously [29], and the workflow is shown in Fig. 1B. Briefly, lentiviral infections were performed to determine the optimal MOI for cas9 lentivirus-infected cells. Twenty-four hours after infection, the cells were selected in puromycin for one week to generate a mutant cell pool, which then continued to be treated with DMSO (vehicle, Day 0) and lenvatinib (lenvatinib concentration: 1000 nM; Fig. 1C). After 21 days, lenvatinib-resistant cells were enriched (Day 21), and then cells of Day 0 and Day 21 were collected for genomic DNA extraction. Genomic DNA was extracted using the Qiagen Blood and Cell Culture DNA Maxi Kit according to the manufacturer’s protocol. The sgRNA sequences were PCR-amplified and adapted for Illumina sequencing (Illumina, San Diego, CA, USA). Sequencing and analysis of genome-scale screens followed, as described before [29, 30], and all data sets were processed in R (currently v3.4.1) to produce bioinformatic analysis-ready image files.

### Establishment of knockout and knockdown cell lines

The construction of sgRNA expression plasmids was performed as described previously [31]. The sequences used to construct the sgRNA-encoding plasmids can be found in Table S1. A list of all used sgRNA oligo sequences can be found in Table S2. shRNA sequences are included in Table S3. The control shRNA, NF1 shRNA, DUSP9 shRNA, DUSP9-overexpressing adenovirus, and all plasmids were constructed by Genomeditech (Shanghai) Co., Ltd. The NF1 gene has a full length of 8457 bp, which makes it impossible to construct a lentiviral vector. The main function of NF1 is transmitted by the GTPase activator protein related domain (GRD), so we chose to construct NF1-GRD as the expression sequence for NF1 overexpression experiments. The expression was confirmed by PCR and western blotting. For rescue experiments, Huh7 and PLC/PRF/5 cells were transfected with PGMLV-H_NF1-GRD-3×Flag-PGK-Neo and PGMLV-CMV-H_DUSP9-PGK-Hygromycin plasmid for the construction of NF1 and DUSP9 overexpression. The efficacy of the transfection was tested by examining the expression of the Flag protein using western blotting.

### Quantitative RT-PCR and western blotting

Total RNA was extracted using Trizol reagent (Thermo, USA) by following their routine procedure. For cDNA synthesis, total RNA was reverse transcribed to cDNA with a SuperScript VILOTM cDNA Synthesis Kit (Thermo, USA) according to the manufacturer’s protocol. The cDNA was amplified using SYBR Green PCR Master Mix (Yeasen, Shanghai) and normalized using the housekeeping gene glyceraldehyde 3-phosphate dehydrogenase (GAPDH). The primers used are listed in S2 Table. Relative expression levels were calculated using the 2^ΔΔCt^ method for qPCR analysis by normalizing to GAPDH. The protein expression of NF1 was determined by western blotting using anti-NF1 antibody produced in rabbits (14623,CST) at a dilution of 1:1000 using the monoclonal anti-β-Actin antibody (Sigma, dilution 1:1000) as a loading control. DUSP9 protein expression was detected using polyclonal antibody (194355, Abcam, 1:5000). The total protein lysate was extracted by RIPA buffer. The antibodies AKT (4691, CST), p‐AKT (4060, CST), ERK (sc-271269, CST), p‐ERK (sc-7383, CST), anti-FOXO3 (2497, CST) and anti-pFOXO3 (5538, CST) were used at 1:1000 dilution, respectively.

### In vitro experiments

Viability and colony formation assays were performed as described above [29]. Briefly, for cell proliferation assay, the transfected cells were seeded into 96-well plates at a density of 2000 cells per well, O.D. absorbance at 450 nm was read, and relative OD versus serial standard curve was determined. For clonogenic assays, 300 cells/well were plated in six-well plates. After a 24 h cell attachment period, the cells were treated with 1000 nM lenvatinib. Control wells in the experiments included 0.5% DMSO. The colonies were fixed with ice-cold methanol, stained with crystal violet (0.5% in 25% methanol) and counted. To determine cell proliferation rates, the indicated cell numbers were seeded in 10 cm dishes, and cell counts were carried out over a total of 6–7 weeks via a hemocytometer. For transwell migration and invasion assays, HCC cells (1 × 10^5^) were seeded into the upper chamber without (transwell migration assay) or with (transwell invasion assay, cells were serum-starved for 6 h prior to the experiment) matrigel (BD Biosciences, USA). 500 µL of 30% FBS containing culture media (with or without lenvatinib) were then added to each well of the 24-well plate. The cells were allowed to migrate and invade for 12 h; they were then stained with 0.1% crystal violet and counted under a microscope. The cell cycle apoptosis and cell cycle distribution were analyzed by flow cytometry according to the manufacturer’s instructions after 72 h of culture. Cell viability was determined using an MTT assay, and IC50 values were calculated as described before [32].

### Animal experiments

Animal experiments in this study were carried out on male 5-week-old BALB/cAnN-nu (nude) mice. For the orthotopic tumor implantation model, Huh7 NF1 knockout cells were first prepared with sgRNA NC cells as controls. The mice were inoculated subcutaneously with 0.1 mL of Huh7 cell suspension (2 × 10^6^ cells) using a sterile 22-gauge needle. On the 8th day after tumor cell inoculation, 36 tumor-bearing nude mice were randomly divided into six groups with five in each group for treatment. Depending on the group, lenvatinib (10 mg/kg/Day) or trametinib (3 mg/kg/Day) was orally administered for 21 days. At the end of each set of experiments, the mice were sacrificed by cervical dislocation; the tumors were excised, and tumor weights and volumes were measured.

### Statistical analysis

All statistical analysis was performed using GraphPad Prism 7 (GraphPad Software Inc., La Jolla, CA, USA). Unless indicated otherwise, data are presented as mean ± SEM (SEM, standard error of the mean) of three independent experiments. A two-tailed *t*-test was used to analyze the difference between two groups, and a one-way ANOVA was used among multiple groups. Bioinformatics analysis was performed using R version 3.6.3 (clusterProfiler package for Go and KEGG analysis; Org.hs.eg.db package, version 3.10.0, for for ID conversion) [33]. Values of *P* < 0.05 were considered statistically significant (*p < 0.05, **p < 0.01, ***p < 0.001).

## Supplementary information

Supplementary materials

## Data Availability

The datasets used and/or analyzed during the current study are available from the corresponding author on reasonable request
