# Correction: Genome-scale CRISPR-Cas9 knockout screening in hepatocellular carcinoma with lenvatinib resistance

**DOI:** 10.1038/s41420-022-00836-6

**Published:** 2022-02-21

**Authors:** Yonggang Lu, Haoming Shen, Wenjie Huang, Sha He, Jianlin Chen, Di Zhang, Yongqi Shen, Yifan Sun

**Affiliations:** 1 Department of Hepatobiliary Surgery, Affiliated Liutie Central Hospital of Guangxi Medical University, Guangxi, China; 2 Department of Clinical Laboratory, Hunan Cancer Hospital & The Affiliated Cancer Hospital of Xiangya School of Medicine, Central South University, Hunan, China; 3 Department of Clinical Laboratory, Affiliated Liutie Central Hospital of Guangxi Medical University, Guangxi, China; 4 Department of Clinical Laboratory, The Third Xiangya Hospital of Central South University, Hunan, China; 5 Department of Oncology, Affiliated Liutie Central Hospital of Guangxi Medical University, Guangxi, China

**Keywords:** Cancer, Biomarkers, Medical research

Correction to: *Cell Death Discov*ery 10.1038/s41420-021-00747-y, published online 18 November 2021

The original version of this article unfortunately contained a mistake. There were errors in Figs. 2b, 5b and 5d. The authors apologize for the error. The original article has been corrected.

