# Supplementary material for: Genome-scale CRISPR-Cas9 knockout screening in hepatocellular carcinoma with lenvatinib resistance
Source: Cell Death Discov. 2021 Nov 18;7:359. doi: 10.1038/s41420-021-00747-y (PMC8602346; doi:10.1038/s41420-021-00747-y)
Supplement: Supplementary file 1 — Supplementary materials [file 41420_2021_747_MOESM1_ESM.pdf]

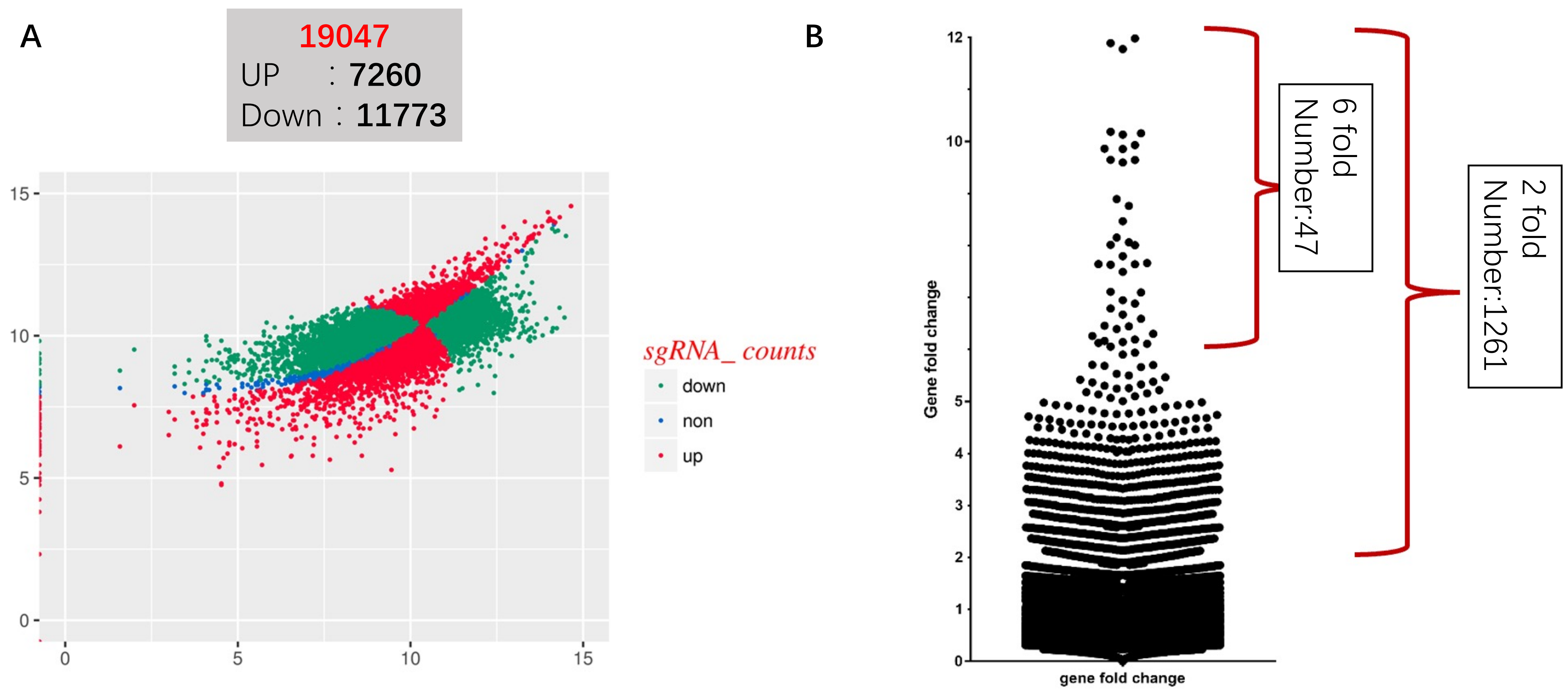

Figure S1. Results from CRISPR/Cas9 knockout library screening. A. 19047 sgRNA were found after high- throughput sequencing analysis; B. Scatter plot to indicate the numbers of sgRNAs with fold-change >2 or 6

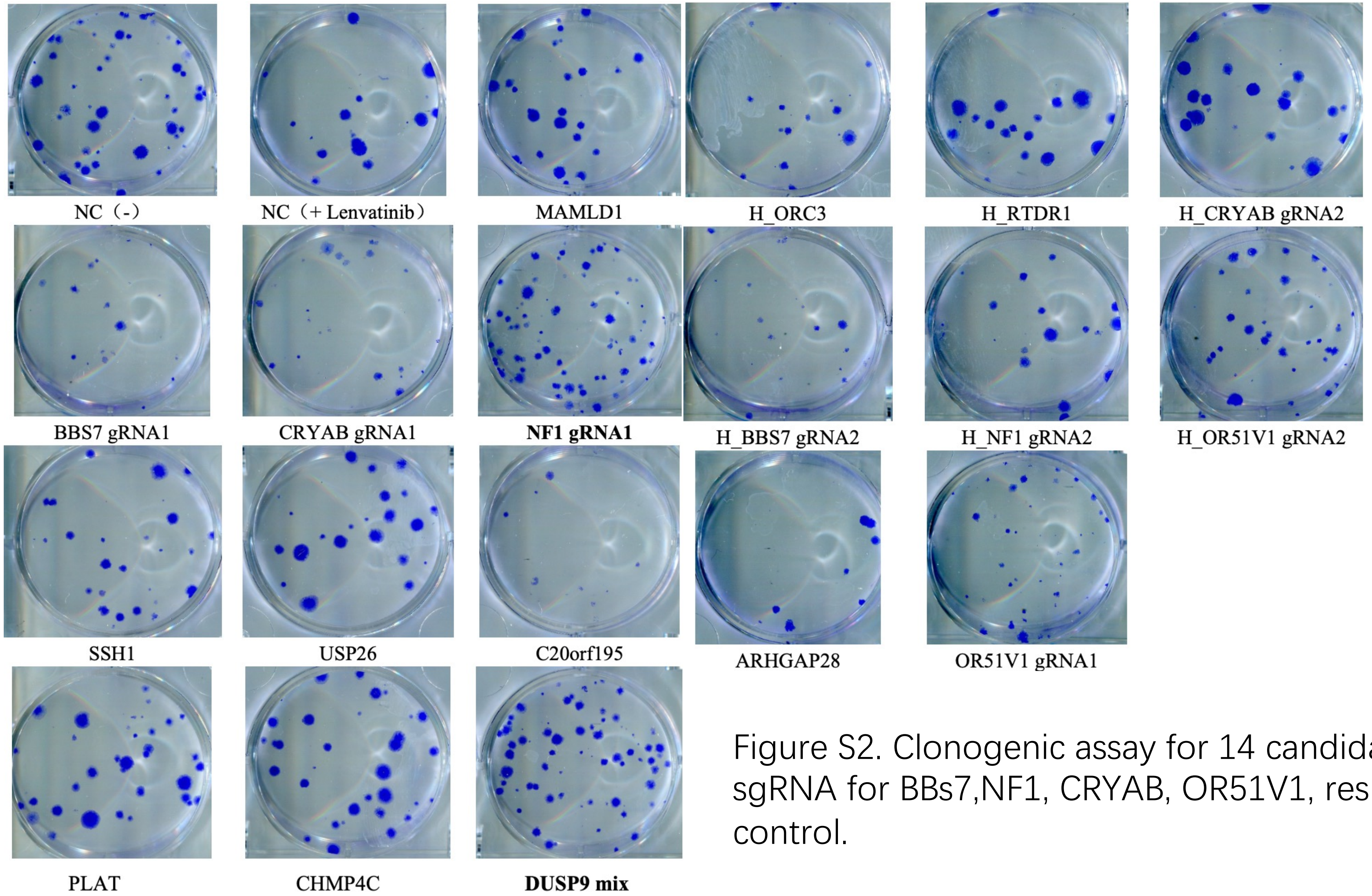

Figure S2. Clonogenic assay for 14 candidate genes. There are two sgRNA for BBs7,NF1, CRYAB, OR51V1, respectively. NC, Negative control.

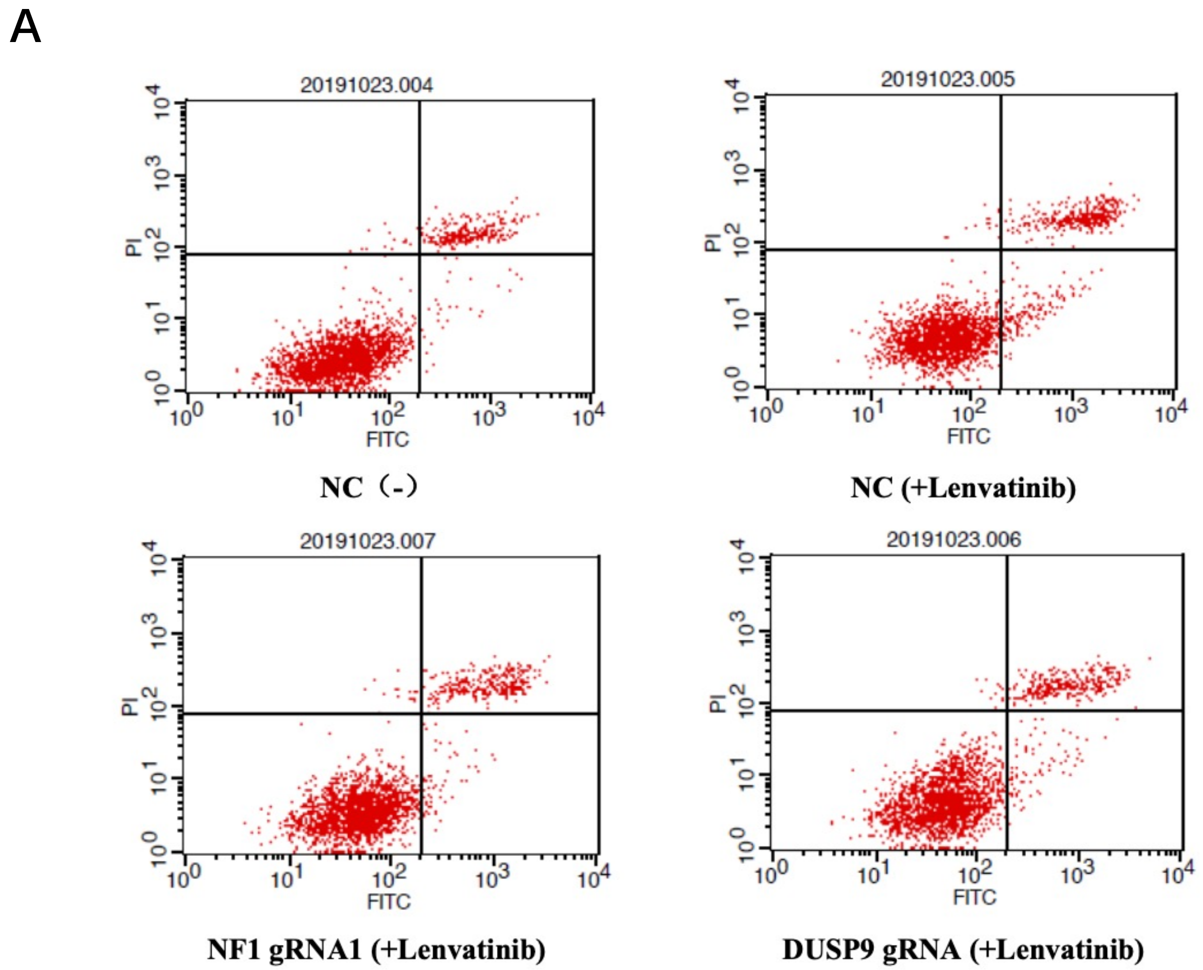

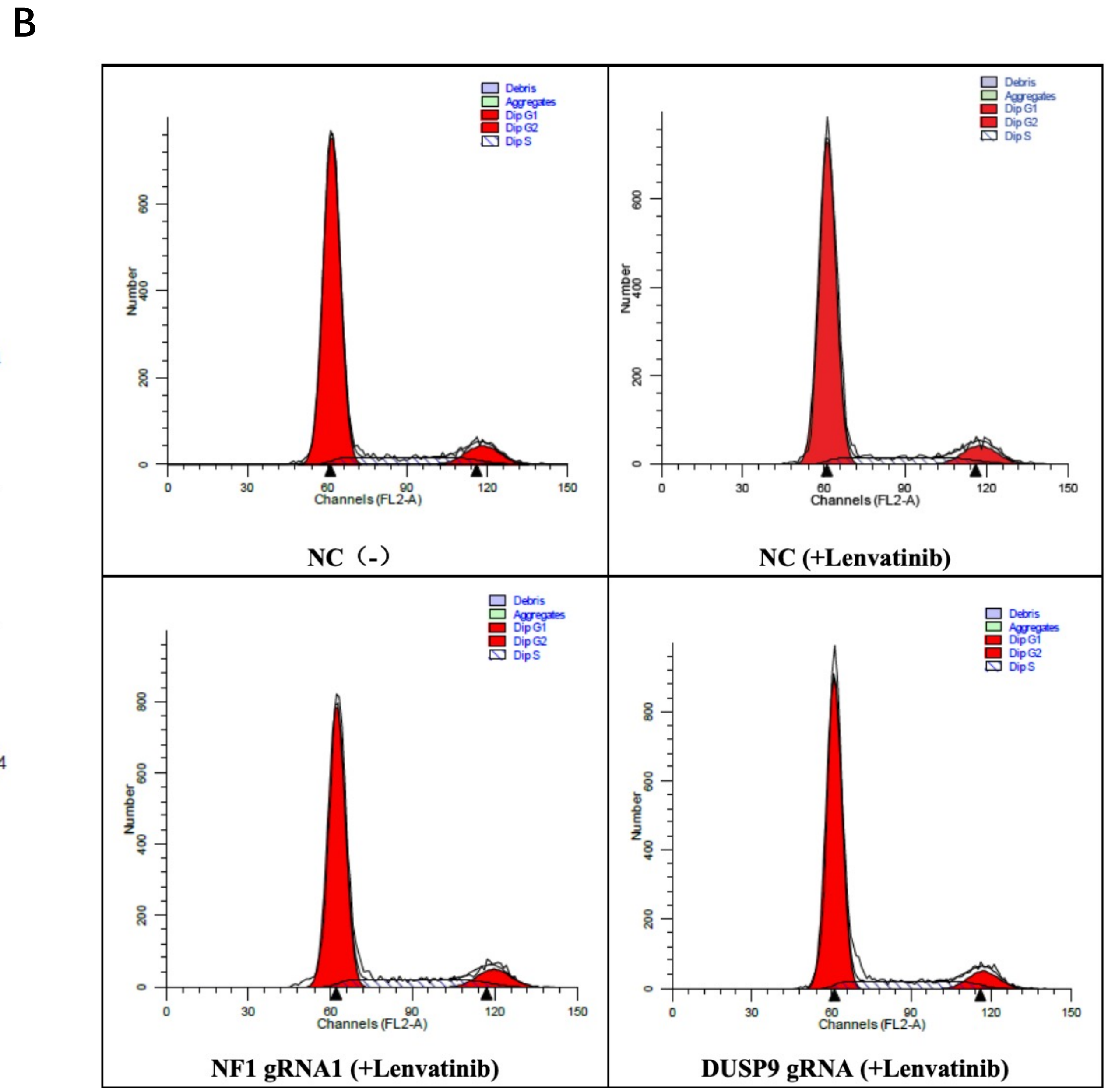

Figure S3. Cell apoptosis(A) and cycle(B) performed by flow citometry

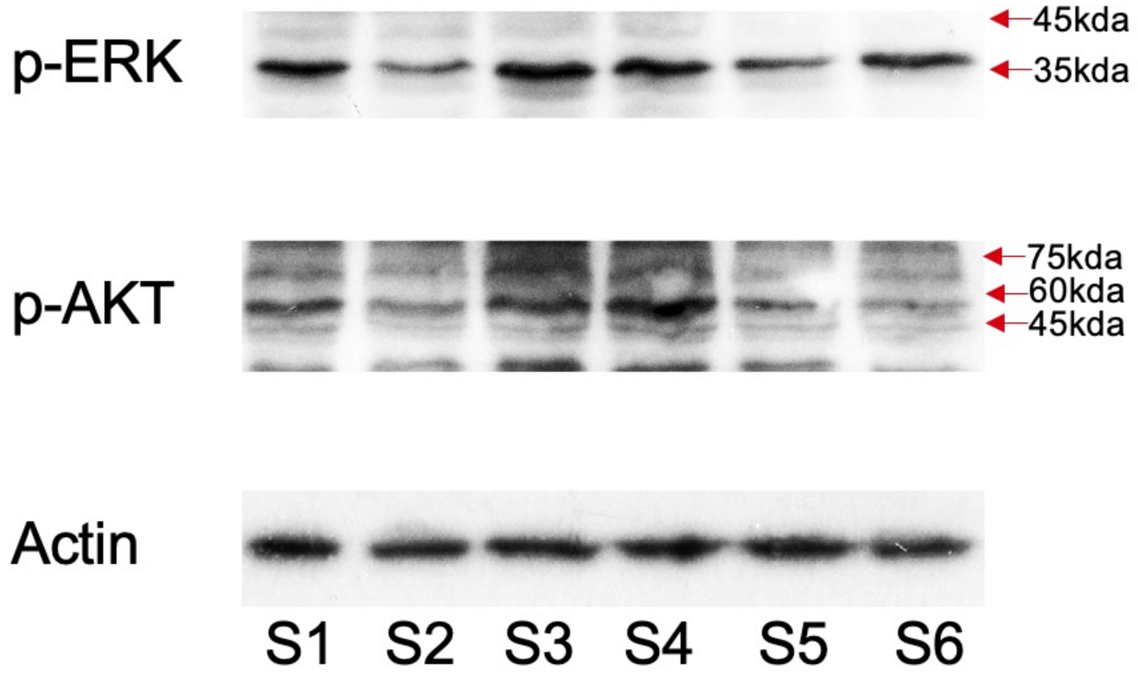

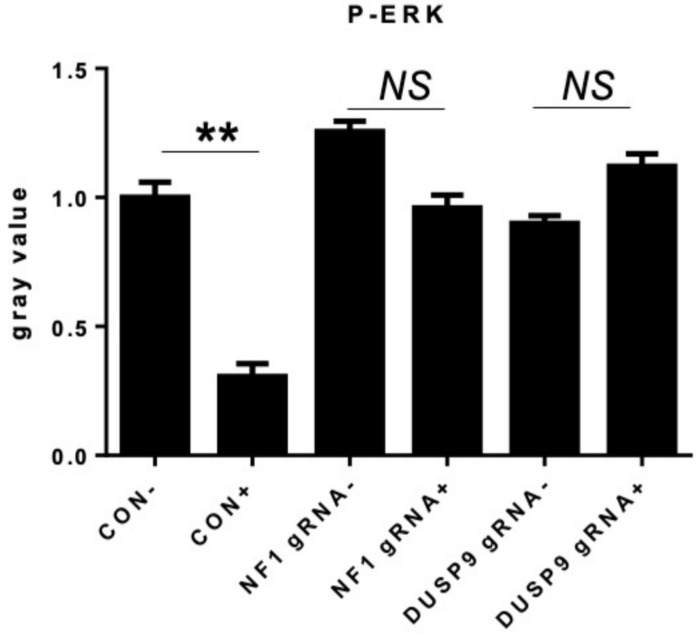

Figure S4. Expression of genes of interest in Huh7 cells was measured by Western blot.
S1: CON- S2：CON+Lenvatinib
S3：NF1 gRNA- S4：NF gRNA+ Lenvatinib
S5：Dusp9 gRNA- S6：DUSP9+ Lenvatinib
Anti-p-ERK and p-AKT (1:2000). Goat anti-rabbit-HRP secondary antibody was applied at 1:3000.

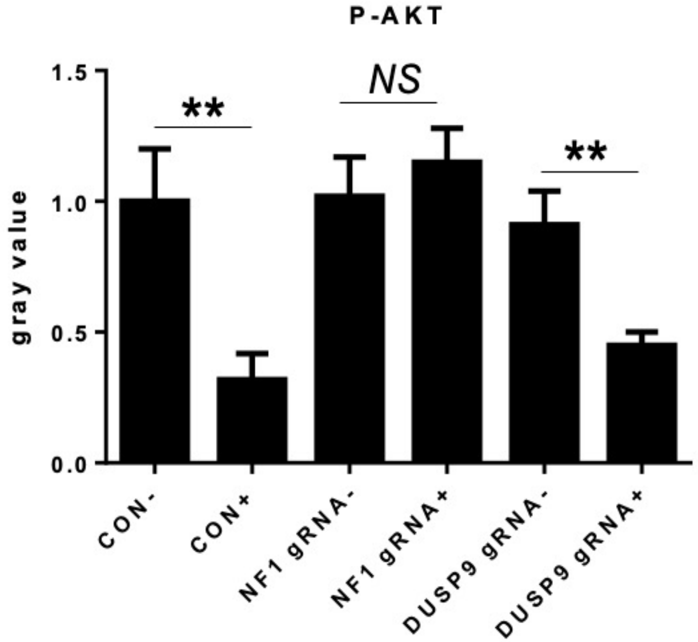

Table S1 The sequences used to construct the sgRNA-encoding plasmids

NO.	TargetSeq
NC	ACGGAGGCTAAGCGTCGCAA
H_ARHGAP28	AATGTTCAGAAAACCAGATT
H_BBS7	TTGTGTAGCTCTGTGTCTTG
H_BBS7	AAGACACAGAGCTACACAAA
H_C20orf195	CAACTCATACTGCTCCGATG
H_CHMP4C	AAACAGCTCACTCAGATTGA
H_CRYAB	TTTCTAGATGCGCCTGGAGA
H_CRYAB	GTGGTGGATGGCGATGTCCA
H_DUSP9	TCTCAACGATGCCTATGACC
H_DUSP9	GTGTGGCACCCTCCGAATCC
H_MAMLD1	ATCTTCTTCCTCCATCCACG
H_NF1	GTTGTGCTCAGTACTGACTT
H_NF1	AGTCAGTACTGAGCACAACA
H_OR51V1	CTTCTCCTCAATCTATGCCA
H_OR51V1	TAGTTACTATGCCCTGATGC
H_ORC3	TTTCTGCAAAAATCACATTC
H_PLAT	CTCCTCTTCTGAATCGGGCA
H_RTDR1	CAACCAGAACATCCGCAGCA
H_SSH1	ATTTAAGCCTGTGTCTGTCC
H_USP26	TCATGCATCATGAACGCCAC

NC, Negative control.

Table S2 The sgRNA oligo sequences for candidate genes

Plasmid's names	Primer Top	Primer Bottom
NC gRNA	caccgACGGAGGCTAAGCGTCGCAA	aaacTTGCGACGCTTAGCCTCCGTc
H_CRYAB gRNA	caccgTTTCTAGATGCGCCTGGAGA	aaacTCTCCAGGCGCATCTAGAAAc
H_ARHGAP28 gRNA	caccgAATGTTCAGAAAACCAGATT	aaacAATCTGGTTTTCTGAACATTc
H_BBS7 gRNA	caccgTTGTGTAGCTCTGTGTCTTG	aaacCAAGACACAGAGCTACACAAc
H_BBS7 gRNA2	caccgAAGACACAGAGCTACACAAA	aaacTTTGTGTAGCTCTGTGTCTTc
H_C20orf195 gRNA	caccgCAACTCATACTGCTCCGATG	aaacCATCGGAGCAGTATGAGTTGc
H_CHMP4C gRNA	caccgAAACAGCTCACTCAGATTGA	aaacTCAATCTGAGTGAGCTGTTTc
H_CRYAB gRNA2	caccGTGGTGGATGGCGATGTCCA	aaacTGGACATCGCCATCCACCAC
H_DUSP9 gRNA	caccgTCTCAACGATGCCTATGACC	aaacGGTCATAGGCATCGTTGAGAc
H_DUSP9 gRNA2	caccGTGTGGCACCCTCCGAATCC	aaacGGATTCGGAGGGTGCCACAC
H_MAMLD1 gRNA	caccgATCTTCTTCCTCCATCCACG	aaacCGTGGATGGAGGAAGAAGATc
H_NF1 gRNA	caccGTTGTGCTCAGTACTGACTT	aaacAAGTCAGTACTGAGCACAAC
H_NF1 gRNA2	caccgAGTCAGTACTGAGCACAACA	aaacTGTTGTGCTCAGTACTGACTc
H_OR51V1 gRNA	caccgCTTCTCCTCAATCTATGCCA	aaacTGGCATAGATTGAGGAGAAGc
H_OR51V1 gRNA2	caccgTAGTTACTATGCCCTGATGC	aaacGCATCAGGGCATAGTAACTAc
H_ORC3 gRNA	caccgTTTCTGCAAAAATCACATTC	aaacGAATGTGATTTTTGCAGAAAc
H_PLAT gRNA	caccgCTCCTCTTCTGAATCGGGCA	aaacTGCCCGATTCAGAAGAGGAGc
H_RTDR1 gRNA	caccgCAACCAGAACATCCGCAGCA	aaacTGCTGCGGATGTTCTGGTTGc
H_SSH1 gRNA	caccgATTTAAGCCTGTGTCTGTCC	aaacGGACAGACACAGGCTTAAATc
H_USP26 gRNA	caccgTCATGCATCATGAACGCCAC	aaacGTGGCGTTCATGATGCATGAc

There are two sgRNA for BBs7,NF1, CRYAB, OR51V1, respectively

Table S3 shRNA sequences for NF1 and DUSP9

Name	sequences 5'to 3'
Primer-NC-T	gatctGTTCTCCGAACGTGTCACGTTTCAAGAGAACGTGACACGTTCGGAGAATTTTTTc
Primer-NC-B	aattgAAAAAATTCTCCGAACGTGTCACGTTCTCTTGAAACGTGACACGTTCGGAGAACa
H_NF1-shRNA1-T	gatccGCCAACCTTAACCTTTCTAATCTCGAGATTAGAAAGGTTAAGGTTGGCTTTTTT
H_NF1-shRNA1-B	aattAAAAAAGCCAACCTTAACCTTTCTAATCTCGAGATTAGAAAGGTTAAGGTTGGCg
H_NF1-shRNA2-T	gatccGCTGGCAGTTTCAAACGTAATCTCGAGATTACGTTTGAAACTGCCAGCTTTTTT
H_NF1-shRNA2-B	aattAAAAAAGCTGGCAGTTTCAAACGTAATCTCGAGATTACGTTTGAAACTGCCAGCg
H_dusp9-shRNA1-T	gatccGCATCCGCTACATCCTCAATGCTCGAGCATTGAGGATGTAGCGGATGCTTTTTT
H_dusp9-shRNA1-B	aattAAAAAAGCATCCGCTACATCCTCAATGCTCGAGCATTGAGGATGTAGCGGATGCg
H_dusp9-shRNA2-T	gatccGGCCATTGAGTTCATTGATGACTCGAGTCATCAATGAACTCAATGGCCTTTTTT
H_dusp9-shRNA2-B	aattAAAAAAGGCCATTGAGTTCATTGATGACTCGAGTCATCAATGAACTCAATGGCCg
H_dusp9-shRNA3-T	gatccGATCCTGCCCAACCTCTATCTCTCGAGAGATAGAGGTTGGGCAGGATCTTTTTT
H_dusp9-shRNA3-B	aattAAAAAAGATCCTGCCCAACCTCTATCTCTCGAGAGATAGAGGTTGGGCAGGATCg

T: Top; B: Bottom

Table S4. The top 10 for BP and CC in Go analysis

ONTOLOGY	ID	Description	pvalue	p.adjust	qvalue
BP	GO:0060541	respiratory system development	6.9541E-07	0.00306249	0.00277695
BP	GO:0048880	sensory system development	1.3248E-06	0.00306249	0.00277695
BP	GO:0032944	regulation of mononuclear cell proliferation	1.9248E-06	0.00306249	0.00277695
BP	GO:0098742	cell-cell adhesion via plasma-membrane adhesion molecules	2.4122E-06	0.00306249	0.00277695
BP	GO:0050670	regulation of lymphocyte proliferation	2.883E-06	0.00306249	0.00277695
BP	GO:0150063	visual system development	3.8831E-06	0.00306249	0.00277695
BP	GO:0031341	regulation of cell killing	4.0764E-06	0.00306249	0.00277695
BP	GO:0070663	regulation of leukocyte proliferation	4.2167E-06	0.00306249	0.00277695
BP	GO:0032946	positive regulation of mononuclear cell proliferation	4.2772E-06	0.00306249	0.00277695
BP	GO:0007156	homophilic cell adhesion via plasma membrane adhesion molecules	5.2341E-06	0.00317256	0.00287675
CC	GO:0030667	secretory granule membrane	5.6659E-05	0.03571522	0.03277385
CC	GO:0062023	collagen-containing extracellular matrix	9.3201E-05	0.03571522	0.03277385
CC	GO:0005788	endoplasmic reticulum lumen	0.00015371	0.03571522	0.03277385
CC	GO:0031093	platelet alpha granule lumen	0.00026204	0.03571522	0.03277385
CC	GO:0031091	platelet alpha granule	0.00026468	0.03571522	0.03277385
CC	GO:0016323	basolateral plasma membrane	0.00031489	0.03571522	0.03277385
CC	GO:0016605	PML body	0.00032384	0.03571522	0.03277385
CC	GO:0045177	apical part of cell	0.00044774	0.04320684	0.03964849
CC	GO:0005667	transcription factor complex	0.00098637	0.08274313	0.07592872
CC	GO:1990907	beta-catenin-TCF complex	0.00111362	0.08274313	0.07592872
